# Utility of non-contrast-enhanced magnetic resonance imaging in predicting preoperative clinical stage and prognosis in patients with thymic epithelial tumor

**DOI:** 10.1007/s11604-022-01358-y

**Published:** 2022-11-14

**Authors:** Asako Kuhara, Akiko Sumi, Tomonori Chikasue, Atsushi Kawaguchi, Shuichi Tanoue, Shuji Nagata, Masamichi Koganemaru, Toshi Abe, Masaki Kashihara, Masahiro Mitsuoka, Hidenobu Ishii, Koichi Ohshima, Ann N. C. Leung, Kiminori Fujimoto

**Affiliations:** 1 Department of Radiology, Kurume University School of Medicine, 67 Asahi-Machi, Fukuoka, Kurume, 830-0011 Japan; 2 Education and Research Center for Community Medicine, Faculty of Medicine, Saga University, Saga, Japan; 3 Department of Surgery, Kurume University School of Medicine, Kurume, Japan; 4 Division of Respirology, Neurology, and Rheumatology, Department of Internal Medicine, Kurume University School of Medicine, Kurume, Japan; 5 Department of Pathology, Kurume University School of Medicine, Kurume, Japan; 6 Department of Radiology, Stanford University, Stanford, CA USA

**Keywords:** Thymic epithelial tumor, Magnetic resonance imaging, Contrast media, World Health Organization Classification, TNM stage

## Abstract

**Purpose:**

The purpose of this study was to find useful imaging features on non-contrast-enhanced magnetic resonance imaging (MRI) that can divide patients with thymic epithelial tumor (TET) into clinical stage I-II and III-IV groups under assumption that contrast media are contraindicated.

**Materials and methods:**

This retrospective study included 106 patients (median age, 60 years; range, 27–82 years; 62 women) with surgically resected TET who underwent MRI between August 1986 and July 2015. All cases were classified according to the 2015 WHO classification and staged using the eighth edition of the TNM system. Two radiologists independently evaluated 14 categories of MRI findings; the findings in patients with stage I-II were compared with those of patients with stage III-IV using a logistic regression model. Disease-specific survival associated with significant findings was calculated using the Kaplan–Meier method.

**Results:**

Univariate analysis showed that stage III-IV patients were more likely to have tumors with an irregular contour, heterogeneity on T1WI, low-signal intensity on T2WI, irregular border with lung, findings of great vessel invasion (GVI) (hereafter, GVI sign), pericardial thickening/nodule, and lymphadenopathy (all, *P* < 0.01). On multivariable analysis, only two findings, irregular border between tumor and lung (odds ratio [OR], 272.8; 95% CI 26.6–2794.1; *P* < 0.001) and positive GVI sign (OR, 49.3; 95% CI 4.5–539.8; *P* = 0.001) remained statistically significant. Patients with one or both features had significantly worse survival (log-rank test, *P* < 0.001).

**Conclusion:**

For patients with TET who are unable to receive contrast for preoperative staging, the two image findings of an irregular border between tumor and lung and the positive GVI sign on non-contrast-enhanced MRI could be helpful in determining stage III-IV disease which is associated with a worse survival.

## Introduction

Thymic epithelial tumors (TETs) are a rare but well-established group of organ-specific neoplasms of varying malignant potential that includes thymomas, thymic carcinomas, and thymic neuroendocrine tumors [1–3]. Staging of TET has traditionally been performed using the Masaoka classification, which was first proposed in 1981, modified in 1994 (Masaoka-Koga classification), and has been shown to be strongly predictive of overall survival [1, 3–7]. In 2014, a new TNM system for staging of TET was proposed by the International Association for the Study of Lung Cancer Staging and Prognostic Factors Committee and the International Thymic Malignancy Interest Group (IASLC/ITMIG) [6] that has been widely adopted. In this TNM system, resectable TETs are classified stage I or II and are associated with a better prognosis than stage III or IV [6, 8, 9].

The Japan Lung Cancer Society (JLCS) Medical Practice Guidelines for Thymic Tumors [10] recommends the use of contrast-enhanced (CE) chest computed tomography (CT) for detection, differential diagnosis, and staging of anterior mediastinal masses with an option for chest magnetic resonance imaging (MRI) if iodinated contrast is contraindicated. In patients with TET and history of severe allergy to contrast or renal failure, preoperative TNM staging by non-CE MRI may be more accurate than non-CE CT due to its superior contrast resolution, but the role of non-CE MRI has not been sufficiently evaluated in the literature. The purpose of this study was to find useful morphological features on non-CE MRI that can divide patients with TET into clinical stage I or II (stage I-II) and III or IV (stage III-IV) groups under assumption that contrast media are contraindicated.

## Materials and methods

### Patients and histopathologic evaluation

We retrospectively reviewed the medical records of patients who had undergone MRI for evaluation of anterior mediastinal tumors at our hospital from August 1986 to July 2015. We selected all patients who met the following inclusion criteria: (a) surgical resection of pathologically proven TET and (b) MRI performed within 6 weeks of surgery. The Ethics Committee of Kurume University (approval no.18139) approved this retrospective study and waived the requirement for informed consent. Clinical data and surgical data as well as patients’ outcomes were obtained from medical records.

All histopathological specimens were reviewed and classified according to the 2015 World Health Organization (WHO) system by two experienced pathologists (4 and 31 years of experience in pathology) in consensus [1]. This pathological classification includes five subtypes of thymomas (types A, AB, B1, B2, and B3), thymic carcinomas, and neuroendocrine tumors, which are based on the morphology of epithelial cells and on the lymphocyte-to-epithelial cell ratio [1, 7]. The WHO classification was further subdivided into low-risk thymomas (types A, AB, and B1), high-risk thymomas (types B2 and B3), and thymic carcinomas (including neuroendocrine tumors). Based upon clinical, imaging, and pathological data, all cases were staged according to the TNM staging system by a multidisciplinary committee.

### Image acquisition and analysis

MRI was performed on a variety of MR scanners (3.0-T, 1.5-T, and 0.5-T superconducting MR scanners). Transverse T1-weighted spin/fast-spin echo images (repetition time ms/echo time ms 410–940/7–25) and T2-weighted spin/fast-spin echo images (1000–3950/80–100) were obtained in all patients, using the following scan parameters: section thickening, 4–10 mm; intersection gap, 2 mm; field of view, 20–40 cm; matrix 256 × 192 to 512 × 512. Diffusion-weighted image (DWI) sequences (*b* = 0, 500, and 1000 s/mm^2^ or *b* = 0 and 1000 s/mm^2^) were performed with a single-shot echo-planar sequence. The apparent diffusion coefficient (ADC) maps were generated from the DWI images using *b* = 0 and 1000 s/mm^2^ sequences for all cases. The MRI range was limited to the area of mediastinal tumor and not the entire thorax. Since this study assumed patients were unable to receive contrast, CE MRI data were not used.

Two board-certified radiologists (12 and 14 years of experience) blinded to clinical and pathologic data independently reviewed the MRI studies and evaluated 14 tumor features, including size, contour, presence of capsule and septum, signal intensity on T1-weighted images, presence of low-signal intensity on T2-weighted images, homogeneity on T1- and T2-weighted images, border with lung, signs of suspected great vessel invasion (GVI) (hereafter, GVI sign), pericardial thickening/nodule, pleural thickening/nodule, lymphadenopathy, and mean ADC values. Tumor size and ADC value were divided into two categories using median values. After assessing the interobserver agreement, the final decision was made by consensus with participation of a third radiologist (32 years of experience).

**Fig. 1:**
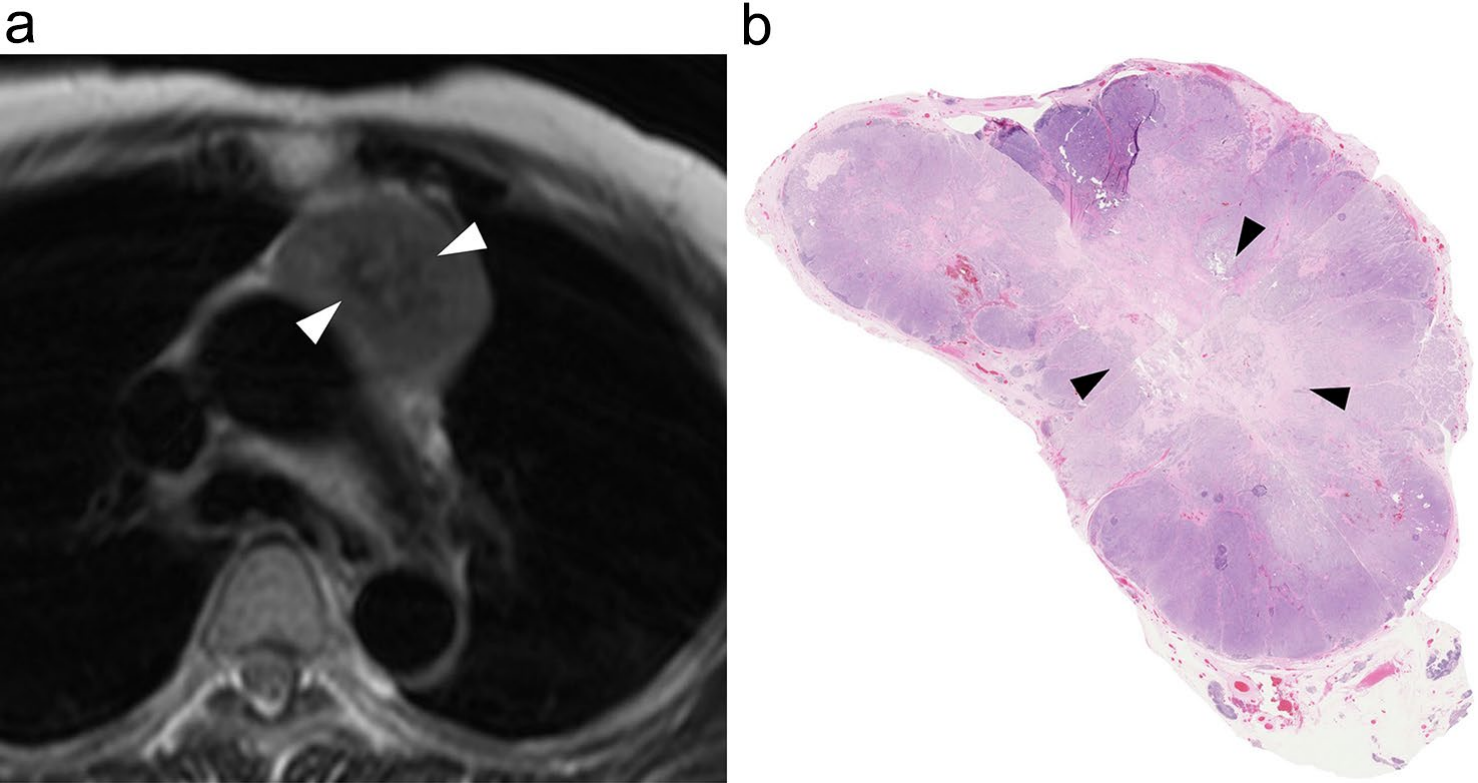
Low-signal intensity within the tumor on T2-weighted image in a 67-year-old woman with thymic squamous cell carcinoma. **a** T2-weighted MR image shows a heterogeneous anterior mediastinal tumor with low-signal intensity (arrowheads). **b** Loupe image of hematoxylin–eosin stain shows the tumor having area of hyaline degeneration (arrowheads)

**Fig. 2:**
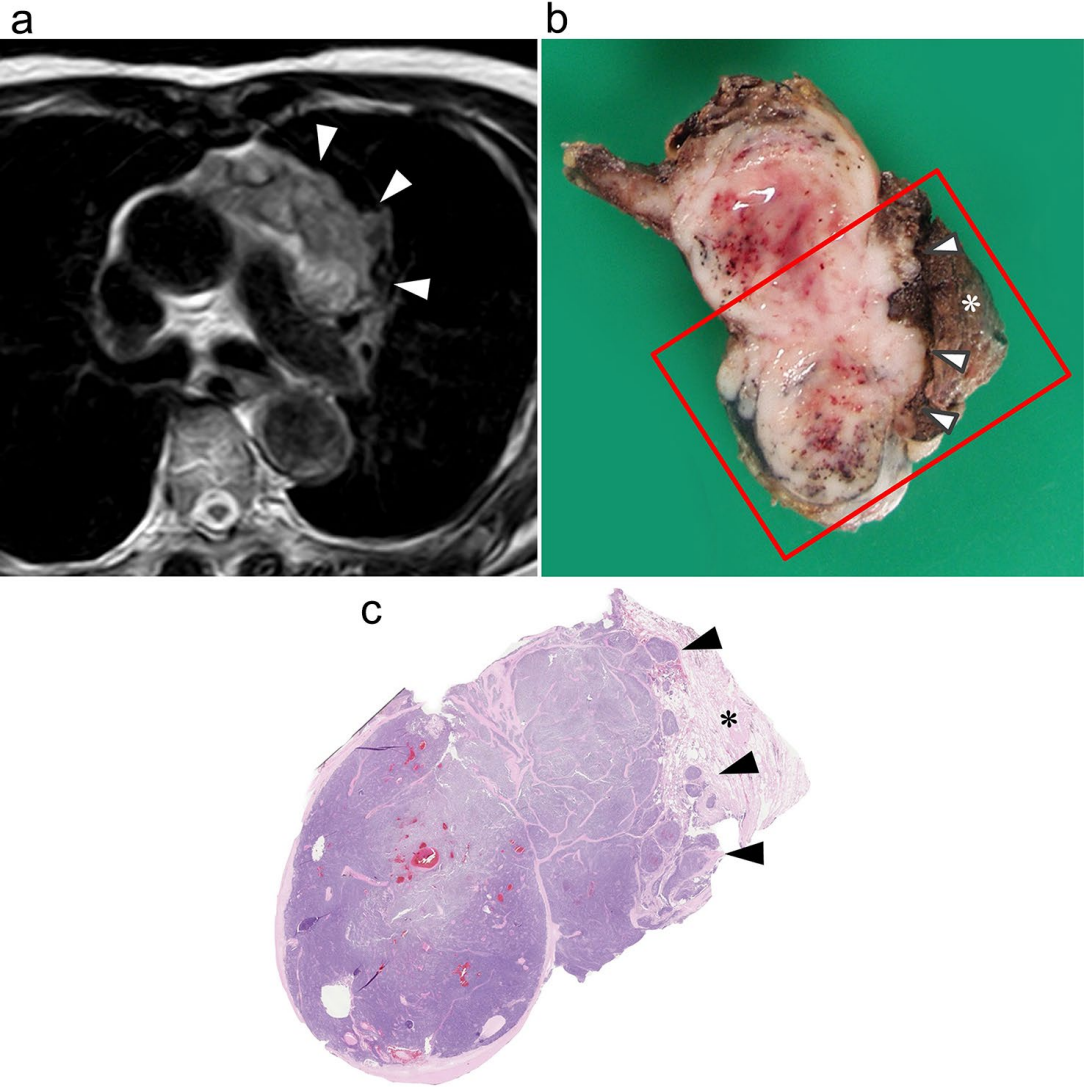
Irregular border between tumor and lung in an 82-year-old man with type B1 thymoma. **a** T2-weighted MR image shows irregular border between anterior mediastinal tumor and lung (arrowheads). **b** Grossly, the tumor is white to yellowish in color and has irregular border (arrowheads) with lung (asterisk). **c** Loupe image of hematoxylin–eosin stain shows the tumor invading (arrowheads) into surrounding lung parenchyma (asterisk)

**Fig. 3:**
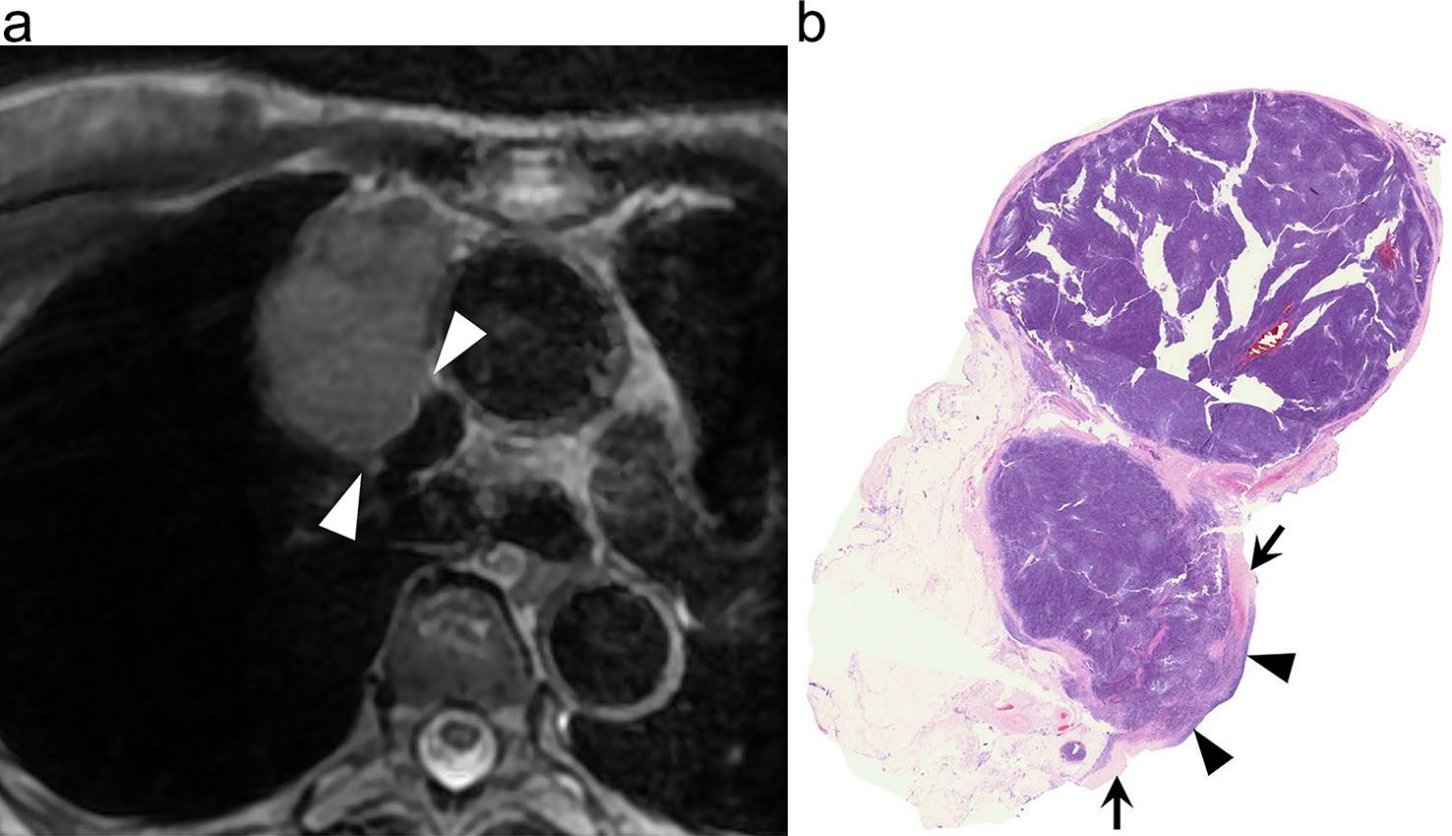
Great vessel invasion (GVI) sign in a 68-year-old woman with type B1 thymoma. **a** T2-weighted MR image shows an anterior mediastinal mass abutting and altering the contour (arrowheads) of the superior vena cava. **b** Loupe image of hematoxylin–eosin stain shows the tumor infiltration of wall (arrowheads) of superior vena cava (SVC). Arrows indicate normal wall of SVC

**Fig. 4:**
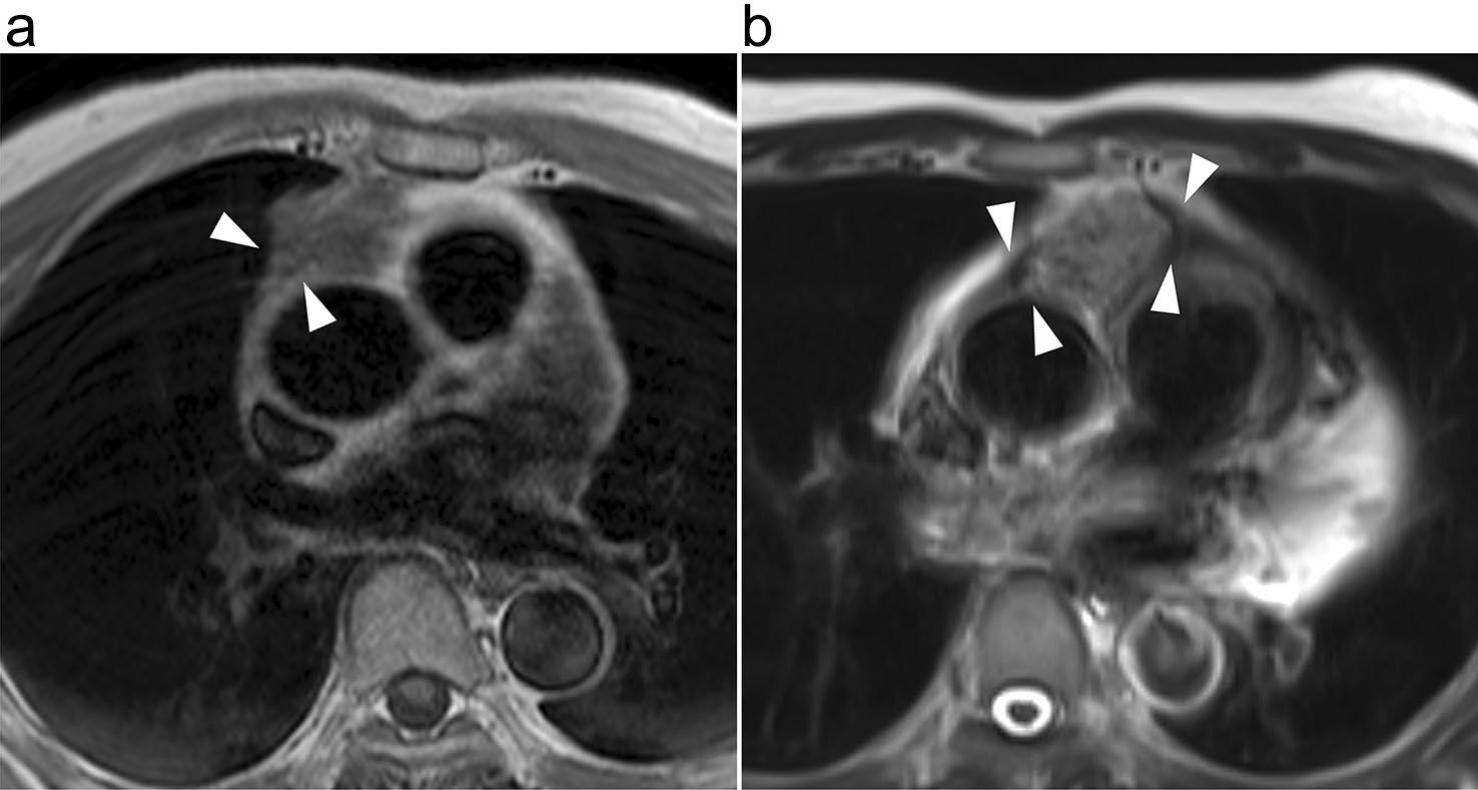
The presence of pericardial thickness/nodule in two patients. **a** 57-year-old man with thymic squamous cell carcinoma. T1-weighted MR image shows an anterior mediastinal tumor with thickened pericardium (arrowheads). **b** 63-year-old man with thymic adenocarcinoma. T2-weighted MR image shows an anterior mediastinal tumor directly in contact with and thickening the pericardium (arrowheads)

Tumor size was defined as the longest diameter measured in any plane [1]. The contour of the whole tumor was determined to be either smooth or lobulated/irregular. The presence of capsule was defined as the presence of low-intensity rim (2 mm or less) along more than two-thirds of the perimeter of the tumor. The presence of septum was defined as a linear network of low- or high-signal intensity dividing the tumor into lobules [2]. The signal intensity of the tumor on T1-weighted image was categorized as low-/iso- or high-signal intensity as compared to that of intercostal muscle. The signal intensity on T2-weighted image was categorized as absence or presence of low-signal intensity within the tumor, again as compared to that of intercostal muscle [2, 11] (Fig. 1). The homogeneity of the tumor was recorded as homogeneous or heterogeneous signal intensity on T1- and T2-weighted images. The border between tumor and lung was divided into non-contact/smooth and irregular (Fig. 2). A positive GVI sign was defined as tumor contiguous to and causing mass effect on vessel, overt endovascular filling defect or occlusion, or signal abnormality of vessel wall [2, 12] (Fig. 3). Pericardial thickening/nodule was defined as present when pericardial thickness was 4 mm or more and the tumor was in direct contact with the pericardium [13–15] (Fig. 4). Pleural thickening/nodule was defined as present when pleural thickening/nodule was present at a site distant from the thymic tumor. Lymphadenopathy was defined as node(s) with a short axis diameter of 10 mm or more. For the measurement of ADC values, one radiologist placed the region of interest (ROI) manually on the entire mass using the slice plane that yielded the largest diameter of the tumor excluding hemorrhagic and cystic components.

### Statistical analysis

Continuous valuables were expressed as the median and range from the 25th to 75th percentile of the interquartile range (IQR). The Mann–Whitney *U* test was used to assess the continuous valuables.

The agreement between the two independent observers was assessed using the kappa statics for the 14 categories of MRI findings mentioned above. Kappa values larger than zero were considered to indicate a positive correlation and the agreement was rated as follows: poor (0.00–0.20), fair (0.21–0.40), moderate (0.41–0.60), good (0.61–0.80), or excellent (0.81–1.00) [16].

The logistic regression model was used to estimate the probability that a patient had stage III or IV (stage III-IV) TET. The variables for multivariate analysis were selected as univariate analysis with a P value of less than 0.20 and without missing values.

When significant MRI findings for predicting stage III-IV were extracted by multivariate analysis, their performance in detecting invasion into adjacent organ/tissue were calculated as follows: sensitivity, specificity, accuracy, positive predictive value (PPV), negative predictive value (NPV), positive likelihood ratio (PLR), and negative likelihood ratio (NLR).

Disease-specific survival time was calculated from the day of the surgery until death or the last known follow-up day. Disease-specific survival with respect to the significant MRI feature was calculated using the Kaplan–Meier method. A P value of less than 0.05 was considered to indicate a significant difference. Statistical analyses were performed by using a software (IBM SPSS Statistics, version 24.0, Mackintosh; IBM, NY).

## Results

A total of 167 patients with TETs underwent surgery between August 1986 and July 2015. Of these, 129 patients underwent MRI before surgery or biopsy. Twenty-three patients were excluded for the following reasons: 16 patients who underwent biopsy only and 7 patients who had incomplete clinical information.

Of the 106 patients who met the study inclusion criteria, there were 44 (42%) men and 62 (58%) women (ages ranged from 27 to 82 years [median, 60 years] (Table 1). One hundred patients underwent complete surgical resection; the remaining 6 patients had incomplete resection due to the residual microscopic tumor in mediastinal fat in 3 patients and the residual macroscopic tumor in 3 patients (one each with residual tumor in SVC, lung, and trachea).  The TNM classification was stage I in 82 (77%) patients, stage II in 3 (3%), stage IIIa in 13 (12%), stage IIIb in 1 (1%), stage IVa in 3 (3%), and stage IVb in 4 (4%).

**Table 1:** Patient and tumor characteristics

Characteristics	Stage I-II (*n* = 85)	Stage III-IV (*n* = 21)	*P* values*
Gender (man/woman)	32/53	12/9	0.10
Age	61.0 (53, 68)	57.0 (49, 68)	0.50
Size, Longest diameter (cm)	5.8 (3.8, 7.0)	6.0 (5.0, 8.8)	0.09
WHO histologic classification			
Low-risk thymoma (A/AB/B1)	47 (7/20/20)	5 (2/0/3)	0.001
High-risk thymoma (B2/B3)	27 (23/4)	6 (2/4)	
Thymic carcinoma/NET	11	10	

^*^P values were calculated by using the Chi-square test or the Mann–Whitney *U* test

Continuous values are expressed as median (IQR: 25%tile, 75%tile)

*WHO* World Health Organization, *NET* neuroendocrine tumor

Using the WHO histological classification system, there were 85 thymomas, 17 thymic carcinomas, and 4 neuroendocrine tumors. Thymomas included 52 low-risk thymomas (9 type A [9%], 20 type AB [19%] and 23 type B1 [22%]) and 33 high-risk thymomas (25 type B2 [24%] and 8 type B3 [8%]). Thymic carcinomas included 15 squamous cell carcinomas (14%) and 2 adenocarcinomas (2%). Neuroendocrine tumors included 3 atypical carcinoids (3%) and one small cell carcinoma (1%) (Table 1).

Additional postoperative therapy was given to 19 patients (radiotherapy in 16 patients, chemotherapy in two, and chemoradiation therapy in one). Twelve patients who underwent radiotherapy alone were stage I, five of which were thymic carcinoma/NET, two with low-risk thymoma with extracapsular invasion, two with low-risk thymoma with combined lung resection, and three with high-risk thymoma with microscopically close to tumor margin, invasion of surrounding fat, or combined pericardium and lung resection. Of the remaining seven patients, five were stage III, and two were stage IV with residual macroscopic tumor, including three who received chemotherapy or chemoradiation therapy. Of the stage III patients, three had thymic carcinoma, one had thymic carcinoma with residual macroscopic tumor post-resection, and one had type B1 thymoma with documented lung involvement. 

There was no statistically significant difference in gender, age, or tumor size between stage I-II and stage III-IV groups (Table 1). Low-risk thymomas were more likely to be stage I-II than stage III-IV (*P* = 0.001, χ^2^ test).

ADC values were evaluated in 32 of 106 patients (3.0-T MR scanner in 15 and 1.5-T MR scanner in 17) with two DWI (*b* = 0, 500, and 1000 s/mm2; *b* = 0 and 1000 s/mm2) sequences performed in 22 patients and 10 patients, respectively. There was no significant difference (*P* = 0.56, Mann–Whitney *U* test) in median ADC values of 1.53 (IQR, 1.05) in stage I-II (*n* = 28) and 1.39 (IQR, 0.55) in stage III-IV (*n* = 4) groups.

Agreement of two independent observers in assessment of MRI findings was good to perfect (***k*** values, 0.6 to 1.0) (Table 2). With univariate analysis, stage III-IV tumors were statistically significantly associated with the MRI findings that were reflected not only tumor invasiveness (i.e., lobulated/irregular contour, irregular border with lung, positive GVI sign, or pericardial thickening/nodule) but also intratumor characteristics (absence of septum, low-signal intensity on T2-weighted image, or heterogeneous signal intensity on T1- and T2-weighted images) (All, *P* < 0.05) (Table 2). There were no cases with pleural thickening/nodule.

**Table 2:** Relationship between MRI findings and TNM stage classification

MRI findings	Kappa values	Stage I-II (*n* = 85)	Stage III-IV (*n* = 21)	Odds ratio	95% Confidence interval		*P* values
					Lower	Upper	
Contour [lobulated/irregular]	0.94	32 (38)	17 (81)	7.0	2.2	22.8	< 0.001
Capsule [absence]	0.96	32 (38)	18 (86)	9.9	2.7	36.4	< 0.001
Septum [absence]	0.87	18 (21)	10 (48)	3.4	1.2	9.2	0.01
Signal intensity on T1WI [high]	0.83	8 (9)	4 (19)	2.3	0.6	8.4	0.19
Low-signal intensity within the tumor on T2WI [presence]	0.77	7 (8)	8 (38)	6.9	2.1	22.1	0.002
Homogeneity of tumor on T1WI [heterogeneous]	0.75	33 (39)	17 (81)	6.7	2.1	21.6	0.001
Homogeneity of tumor on T2WI [heterogeneous]	0.83	52 (61)	19 (90)	6.0	1.3	27.6	0.01
Border between tumor and lung [irregular]	0.94	3 (4)	17 (81)	116.2	23.8	567.1	< 0.001
GVI sign [positive]	0.80	5 (6)	10 (48)	14.5	4.2	50.5	< 0.001
Pericardial thickening/nodule [presence]	0.63	3 (4)	12 (57)	36.4	8.6	153.9	< 0.001
Lymphadenopathy [presence]*	1	0 (0)	5 (24)	57.0	5.1	603.8	< 0.001

Univariate analysis for MRI findings with stage I-II/stage III-IV

The numbers in parentheses are percentages

^*^ Haldane correction was used for estimating odds ratio and 95% confidence interval

Low-signal intensity within the tumor on T2-weighted image was present in 15 of 106 (14%) tumors. Of those, 6 (40%) tumors were thymic carcinomas and 4 (27%) were high-risk thymomas. Among stage III-IV tumors with low-signal intensity within the tumor on T2-weighted images, 2 of 8 (25%) were low-risk thymomas, 3 of 8 (38%) were high-risk thymomas, and 3 of 8 (38%) were thymic carcinomas.

Multivariate logistic regression model of the tumor MRI features showed that stage III-IV tumors were significantly associated with an irregular border with lung (odds ratio [OR], 272.8; 95% CI 26.6–2794.1; *P* < 0.001) and a positive GVI sign (OR, 49.3; 95% CI 4.5–539.8; *P* = 0.001) (Table 3).

**Table 3:** Relationship between MRI findings and TNM stage classification

MRI findings	Odds ratio	95% Confidence interval		*P* values
		Lower	Upper	
Border between tumor and lung [irregular]	272.8	26.6	2794.1	< 0.001
GVI sign [positive]	49.3	4.5	539.8	0.001

Multivariate analysis for MRI findings with stage I-II/stage III-IV

Using surgical findings as the reference standard, the diagnostic performance of MRI features to detect invasion of adjacent structures is shown in Table 4. The diagnostic performance rates of lung infiltration and irregular border lung and tumor were 86% for accuracy, and great vascular infiltration and positive GVI sign were 88% for accuracy and 98% for NPV. Pericardial thickening/nodule to identify pericardial invasion was the feature with the highest accuracy (89%) but with a sensitivity of 50% and a specificity of 91%.

**Table 4:** Diagnostic performance of MRI findings in predicting pathological infiltration into adjacent organs

Pathological infiltration predicted by MRI finding	Sensitivity	Specificity	Accuracy	PPV	NPV	PLR	NPR
Lung infiltration predicted by irregular border between tumor and lung	0.73	0.87	0.86	0.40	0.97	5.76	0.31
Great vascular infiltration predicted by positive GVI sign	0.67	0.89	0.88	0.27	0.98	6.00	0.38
Pericardial infiltration predicted by pericardial thickening/nodule	0.50	0.91	0.89	0.27	0.97	5.88	0.55

*PPV* positive predictive value, *NPV* negative predictive value, *PLR* positive likelihood ratio, *NLR* negative likelihood ratio

Pathologically, lymph node metastasis was detected in three cases, of which one case showed lymphadenopathy on MRI. Of the three cases with pulmonary metastasis, only one case was observed pulmonary nodules on MRI. There were no cases with pleural thickening/nodule on MRI; however, two cases had pathological pleural dissemination.

**Fig. 5:**
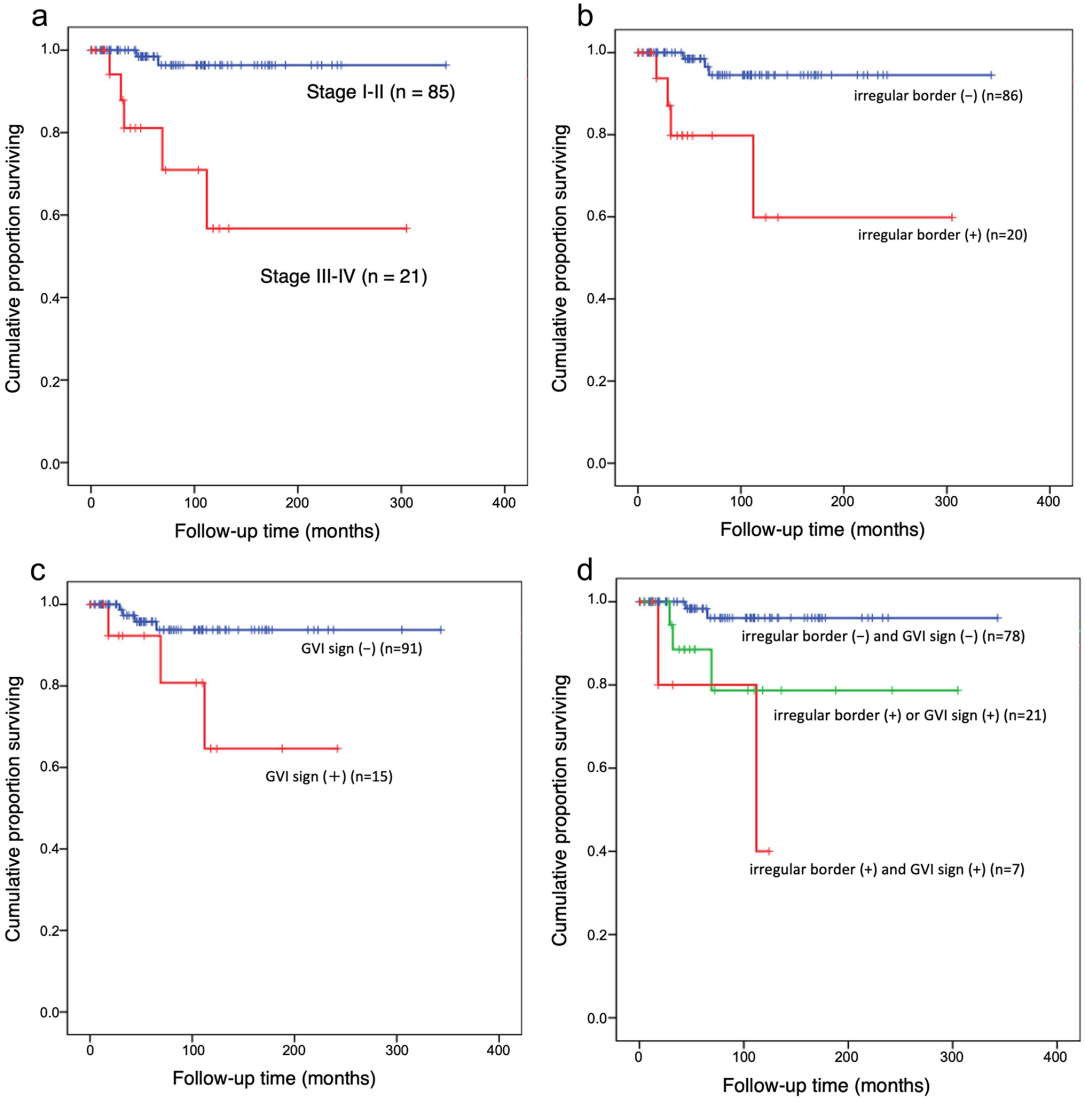
Kaplan–Meier survival curves show disease-specific survival of TNM stage and MRI findings. **a** TNM stage. Patients with stage III-IV had a significantly worse survival than stage I-II patients (log-rank test, *P* < 0.001). **b** Feature of border between tumor and lung. Patients with irregular border between tumor and lung (irregular border ( +)) had a significantly worse survival than those without feature (irregular border (−)) (log-rank test, *P* < 0.001). **c** Feature of GVI sign. Patients with positive GVI sign (GVI sign ( +)) had a significantly worse survival than those without feature (GVI sign (−)) (log-rank test, *P* = 0.03). **d** Features of border between tumor and lung and GVI sign. Patients with one or two features (i.e., irregular border with lung and/or positive GVI sign) (irregular border ( +) and GVI sign ( +)) had significantly worse survival than those without either feature (irregular border (−) and GVI sign (−)) (irregular border ( +) or GVI sign ( +)) (log-rank test, *P* = 0.001)

The median follow-up period was 69 (IQR, 88) months. Seven out of 106 patients (7%) died from TET causes. Patients with stage III-IV had a significantly worse survival than stage I-II patients (log-rank test, *P* < 0.001) (Fig. 5a). Patients with either an irregular border between tumor and lung or a positive GVI sign had significantly worse survival (log-rank test, *P* < 0.001 and *P* = 0.03, respectively) (Fig. 5b, c). There were 7 (7%) patients who had both features, 21 (20%) patients with one feature, and 78 (74%) patients without either feature. Patients with one or both features had a significantly worse survival (log-rank test, *P* = 0.001) (Fig. 5d).

## Discussion

MRI is commonly used to evaluate the internal characteristics or predict the histological diagnosis of tumors [2, 17–19]. In patients with TET, CE CT is usually the diagnostic modality of choice and MRI is not routinely performed; however, a non-CE MRI should be considered for staging and to evaluate invasiveness of the tumor [10, 17] in patients who have contraindications to contrast media due to severe allergy and/or renal failure.

In the current study, an irregular border between tumor and lung and a positive GVI sign were two MRI features found to significantly distinguish between stage I-II and stage III-IV disease and likely reflect the presence of tumor invasion into adjacent structures. In agreement with these results, Xiao et al. performed MRI radiomics analysis for TETs and reported that invasion of adjacent tissue (i.e., peritumoral fat, pleura, pericardium, great vessel, or chest wall) was more common in TNM stage III-IV than stage I-II [8]. In this study, an irregular border between tumor and lung and a positive GVI sign had a relatively low sensitivity and higher specificity in predicting the presence of tumor invasion into adjacent structures; in the presence of these findings, preoperative surgical planning should include likelihood of a complicated resection. Sadohara et al. also reported that thymic carcinomas were associated with a higher prevalence of great vessel invasion than thymomas and that tumor invasion of a great vessel was a significant predictor for poor prognosis [2]. Our results confirm that a positive GVI sign as an important prognostic factor; based upon the detailed MR-pathologic correlation performed in our study, we also found that the NPV of the GVI sign was high. This latter result may provide preoperative information to better inform patient eligibility for surgery.  

In patients with TET, it is known that a successful complete resection greatly improves their prognosis [20], and that the new TNM classification specifically categorizes patients into resectable versus non-resectable groups [6, 21]. From this perspective, evaluation of pericardial infiltration is important for preoperative planning purposes and for prognosis. In the present study, the MRI finding of pericardial thickening/nodule, which predicts pericardial infiltration, showed a statistically significant association with higher stage disease on univariable analysis but not on multivariable analysis. This MRI finding also had higher specificity, accuracy, and NPV as well as other MRI features of tumor invasiveness, such as an irregular border between tumor and lung and a positive GVI sign. On the other hand, a prior study of imaging findings of cardiovascular infiltration in TETs reported that assessment of pericardial infiltration by CINE MRI (sensitivity, 83%) was better than CT (sensitivity, 67%), but both specificities showed 0% as all 12 cases assessed had invasion [22]. Utility of CINE MRI for assessment of pericardial tumor infiltration needs further investigation.

Our results of univariate analysis revealed that MRI findings associated with TNM stage classification were significantly different not only in tumor invasiveness but also in intratumor characteristics (e.g., low-signal intensity within the tumor on T2-weighted image or heterogeneity on T1- and T2-weighted images). Inoue et al. similarly reported significant differences in heterogeneous signal intensity on T1- and T2-weighted images and intratumor low-signal foci on T2-weighted image which may reflect tumor degeneration seen in histologically high-grade TETs, especially thymic carcinoma [11]. Morikawa et al. reported that although the T2 minimum value on histogram analysis did not show significant differences in differentiating histological classification, the T2 minimum values were significantly lower in Masaoka-Koga stage III-IV than stage I-II [23]. Similar to previous reports [2, 11], there was a significant correlation between the TNM classification and the WHO histological classification in the present study. It can be inferred from these results that histologically high-grade TETs tend to have similar MRI findings to those which present at advanced stage. Our study correlated invasiveness and MRI findings, which differs from these previous papers that correlated histological classification with MRI findings [2, 11].  

Of the 32 cases in this study in which ADC values were obtained, there was no significant difference in ADC values between stage I-II and stage III-IV tumors. In contrast to our results, several previous studies have found a significant difference in ADC values of TETs between Masaoka-Koga or Masaoka stage I-II and stage III-IV but differ as to whether the ADC values of stage III-IV tumors are significantly higher [23] or significantly lower [24–26] as compared to stage I-II tumors. Variability in results of these studies may relate to the differences in the placement of the ROI or volume of interest (VOI). Since Morikawa et al. included not only the solid component but also the hemorrhagic, cystic, and necrotic components within the VOIs to assess the ADC values, it is likely that the ADC maximum value would be higher for stage III-IV tumors than for stage I-II tumors [23]. Therefore, evaluation of ADC values requires careful consideration because the results may differ depending on the way placing the ROI or VOI. In addition, advances in MRI technology have made it possible to obtain images with high quality images; however, DWI tends to deteriorate in image quality due to various artifacts, such as chemical shift artifact, magnetic susceptibility artifact, or motion artifact. Thus, the image quality of DWI should also be carefully considered when analyzing ADC values.

Several limitations of this study should be considered. First, the number of cases was small due to the rarity of TETs. Regarding ADC values, some previous papers have shown that Masaoka-Koga or Masaoka stage III-IV tumors had lower ADC values than stage I-II tumors, while others have shown the opposite suggesting that ADC results depends on the method of measurement. Our results showed no significant difference in ADC values between stage I-II and stage III-IV, probably due to the small number of cases in which ADC values could be measured in our study. Future studies investigating the utility of ADC values would optimally include a sufficiently large number of cases that are imaged using a consistent MR protocol. It is hoped that our results will be validated by examining more cases in the future. Second, this study was performed on MR scanners ranging from 0.5 to 3.0-T which may affect the signal characteristics of the tumors. In order to minimize the effect of differences of magnet field strength on tumor characteristics, we evaluated tumor signal intensity relative to muscle which was used as an internal standard and the evaluation of MRI findings was mainly based on morphological findings.  As the agreement of two independent observers on MR features was good to perfect (k values, 0.6 to 1.0), we believe that the effect of differences in MRI scanner on morphological findings was adequately mitigated.  Due to the retrospective nature of our study, MRI studies were limited to the portion of the thorax containing the TET. Given the unsuitability of this limited coverage for comprehensive thoracic staging, our study was designed specifically to evaluate for locoregional findings that may allow identification of more aggressive TETs (stage III-IV). In this setting, adjunctive studies, such as 18F-fluorodeoxyglucose positron emission tomography and/or whole-body non-CE CT, may be needed to look for distant disease.  

In conclusion, for patients with TET who are unable to receive contrast for preoperative staging, the two image findings of an irregular border between tumor and lung and a positive GVI sign on non-CE MRI could be helpful in determining stage III-IV disease which is associated with a worse survival.

## Data Availability

The datasets generated during and/or analysed during the current study are available from the corresponding author on reasonable request.
